# Roles and Applications of Circulating Tumor-Derived RNAs in Sarcoma Patients: A Systematic Review

**DOI:** 10.3390/ijms252111715

**Published:** 2024-10-31

**Authors:** Elena Gabrielli, Maria Beatrice Bocchi, Cristina Giuli, Francesco Farine, Doriana Di Costa, Giulio Maccauro, Raffaele Vitiello

**Affiliations:** 1 Orthopaedics and Traumatology Department, Fondazione Policlinico Universitario Agostino Gemelli IRCCS, 00168 Roma, Italy; elena.gabrielli01@icatt.it (E.G.); mariabeatrice.bocchi01@icatt.it (M.B.B.); cristina.giuli01@icatt.it (C.G.); francesco.farine01@icatt.it (F.F.); doriana.dicosta01@icatt.it (D.D.C.); raffaele.vitiello@guest.policlinicogemelli.it (R.V.); 2 Orthopaedics and Traumatology Department, Università Cattolica del Sacro Cuore, 00168 Roma, Italy; 3 Villa Stuart Casa di Cura, 00135 Roma, Italy

**Keywords:** circulating tumor nucleic acids, ctNAs, circulating tumor RNA, ctRNA, sarcoma, liquid biopsy, Ewing sarcoma, osteosarcoma, soft tissue sarcoma, STS

## Abstract

Sarcomas are a heterogeneous group of malignancies with a high mortality rate. Detection of circulating tumor-derived material, such as circulating RNA in the peripheral blood of patients, has shown to be useful in diagnosis, prediction of prognosis and disease monitoring in several malignancies. This systematic review aims to probe the existing methods for detecting circulating tumor-derived RNAs from patients affected by sarcoma and their possible clinical application. A systematic review of the literature indexed in PubMed was performed. Each article had to analyze circulating RNA in human specimens obtained from liquid biopsies of patients affected by sarcoma. A total of 26 articles were included. We evaluated 1381 patients; 72% were affected by bone sarcoma and 28% by soft tissue sarcoma. By PCR-based methods, all the studies investigated circulating tumor RNA, mostly in the peripheral blood. Nearly half of the authors investigated the tumor expression and/or release of miRNA (42%). Several authors pointed out that circulating tumor-derived RNA has proven to have potential application in a clinical setting for sarcomas. To the best of our knowledge, this is the first review in the literature to attempt to put together data specifically on ctRNA in patients affected by sarcoma.

## 1. Introduction

Sarcomas are a large heterogeneous group of malignancies which develop from mesenchymal cells. They are classified into two main groups: bone sarcoma (BS) and soft tissue sarcoma (STS). Bone sarcomas arise from the bone and mainly include osteosarcoma (OS), Ewing sarcoma (ES) and chondrosarcoma. Soft tissue sarcomas originate from connective tissue such as fat, muscle, tendons and blood vessels. Among them, there are leiomyosarcoma (LMS), liposarcoma (LS), rhabdomyosarcoma (RMS), synovial sarcoma (SS), malignant peripheral nerve sheath tumor (MPNST) and undifferentiated pleomorphic sarcoma (UPS) [1,2]. Sarcomas account for nearly 21% of all solid pediatric malignant tumors and for less than 1% of all solid adult malignant tumors [3]. Although quite rare, sarcomas have a high mortality rate due to delayed diagnosis because they often become clinically evident at advanced stages of disease, above all, STS [4,5,6,7,8]. Nowadays, tissue biopsy represents the gold standard to make a diagnosis and to stage the disease [9]. However, invasiveness, potential complications and inability to capture dynamic information on tumor development are only a few of the limitations of this technique. In fact, tissue biopsy can offer information exclusively with regard to the genetic alteration in a single part of the tumor and is not capable of detecting intratumoral and intertumoral heterogeneity. Moreover, tissue biopsy lacks reproducibility, as, for example, during the follow-up period when it is difficult to make samples repeatedly, and also applicability, as in the case of tumors located in hard-to-biopsy regions [10,11]. All of the above limitations associated with tissue biopsy have led to the development of liquid-based biopsy techniques that may also provide a chance to guide molecularly based therapeutic approaches [12]. Liquid biopsies have several advantages, such as being minimally invasive, easily accessible and free from potential complications, but most importantly reproducible. The information obtainable from liquid biopsy originates from circulating tumor cells (CTCs), circulating tumor nucleic acids (ctNAs), exosomes and metabolites, which are released and circulate in body fluids such as plasma, serum, saliva, cerebral spinal and urine [13]. Cell-free nucleic acids are released into the bloodstream by the primary tumor cells during necrotic and apoptotic processes, and they are metabolized in the liver and in the kidneys. The remaining circulating nucleic acids can be detected and amplified by polymerase chain reaction (PCR)-based detection methodology. Cell-free nucleic acids include circulating tumor DNA (ctDNA) as well as circulating tumor RNA (ctRNA). The latter includes both protein coding messenger RNA (mRNA) and non-coding RNA such as microRNA (miRNA), long non-coding RNA (lncRNA) and circular RNA (circRNA), classified on the basis of their transcript size and structure [14,15]. In the extracellular matrix, ctRNAs primarily serve as signaling molecules in cell-to-cell communication, which is why one of the major advantages of ctRNA analysis is the possibility to profile RNA expression, which in turn provides valuable information about the overexpression of cancer-specific transcripts [16]. For this reason, ctRNAs are emerging as useful biomarkers for early cancer detection, but also to monitor tumor dynamics and response to therapy [17]. MicroRNAs are short RNA molecules which play a role in many normal biological processes, including cell proliferation, differentiation and apoptosis, by negatively regulating target gene expression at a post-translational level, leading to mRNA degradation or translation interruption. A dysregulation in microRNA function has emerged to be key in carcinogenesis, chemoresistance and metastasis formation [18,19]. Long non-coding RNAs are long RNA molecules which are fundamental in cellular homeostasis. They are often upregulated during tumorigenesis and expressed in tissues only in certain conditions [20]. Circular RNAs are very stable RNAs which have a tumor suppressive effect in several types of cancers by modulating the transcription of linear RNAs, interacting with and translating proteins, and acting as a sponge for miRNAs. For the aforementioned reasons, it is clear that they play an important role in tumorigenesis and metastasis formation. Furthermore, they can be used as potential therapeutic targets thanks to their structural stability [21,22]. The main difference between ctDNA and ctRNA is that there is increasing evidence that ctRNAs in general are more stable and last longer in the bloodstream than ctDNAs because they are protected from degradation by their packaging into exosomes, vesicles and micro-vesicles [23]. In conclusion, ctNAs can be used to discriminate malignant from benign tumors, they can be helpful at making a diagnosis at an early stage, but they also enable us to offer a panoramic view of both the primary and the metastatic tumor in real time by monitoring and predicting tumor dynamics and the treatment response. Finally, they can help in the development of personalized therapy based on tumor heterogeneity [24,25,26]. To date, liquid biopsies are used for all of the above-mentioned purposes in solid cancers such as colorectal, breast and lung cancer to name a few [16,27,28,29,30,31]. The present systematic review aims to probe the existing methods for detecting circulating tumor-derived RNAs from patients affected by sarcoma and their possible clinical application.

## 2. Methods

A systematic review of the literature indexed in the PubMed database using the search term “circulating RNA sarcoma” was performed in May 2024. No filters were applied to the search strategy to minimize the number of missed studies. The bibliographies of the selected studies were accurately searched by hand to identify further studies not found during the electronic search. No restrictions were applied concerning the date of publication. The journal’s title, the author’s name or supporting institutions were not masked at any stage. Lastly, no attempt to contact authors in order to obtain individual patient data was made. The preferred reporting items for systematic reviews and meta-analyses (PRISMA) guidelines were followed. This study was not registered; therefore, there is no registration number. This article adheres to the latest preferred reporting items for systematic reviews and meta-analyses statements [32]. To be considered eligible for this review, each article needed to present some inclusion criteria: (1) analyzed ctRNA in human specimens obtained from liquid biopsies; (2) at least a section of the population under study had to be affected by either bone or soft tissue sarcoma; and (3) longitudinal studies: prospective and retrospective. Furthermore, only full-text articles in the English language were considered. Review articles, cadaveric and animal studies, and non-cohort were excluded.

Initially, the screening was carried out based on titles and abstracts, then the relevant and qualified ones were read in full text, evaluated and selected by three independent authors (D.D.C.; C.G.; F.F.). In case of doubt regarding the inclusion of an article, a senior author made the final decision. Moreover, the methodological quality of the selected studies was assessed with the Methodological Index for Non-Randomized Studies (MINORS) [33]. In the MINORS score, there are 8 items for the non-comparative studies and 12 items for comparative studies. For each item assessed, there is a maximum score of 2. The global score is 16 for non-comparative studies and 24 for comparative studies.

**Figure 1:**
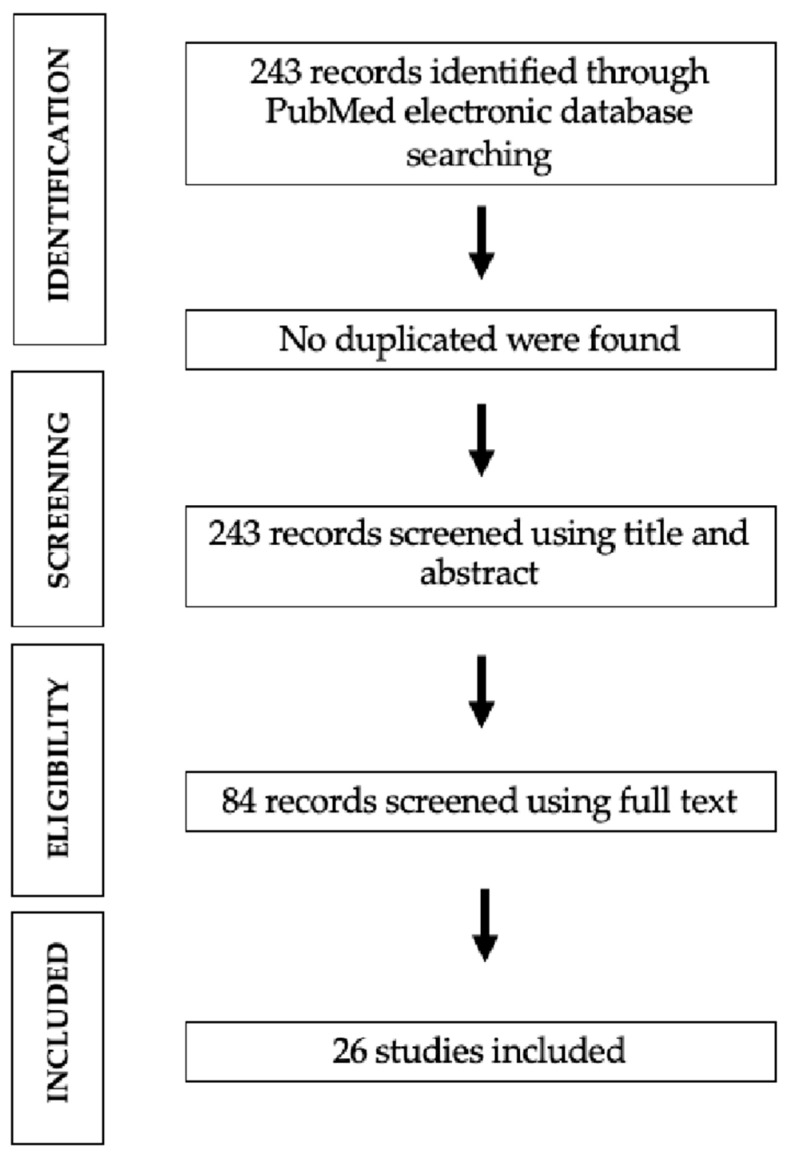
PRISMA flow chart.

Data extraction was carried out to collect information on study design, patient characteristics, methodology and findings. All the selected studies were retrospectively analyzed by three authors (D.D.C; C.G.; F.F.) and inserted into an Excel worksheet (Microsoft Corporation, Redmond, WA, USA. Microsoft Excel [Internet]. 2018. Available from: https://office.microsoft.com/excel). All data collected were categorized by author(s) and year of publication; study design; and patient characteristics, including the total number of patients, the mean age, the type of sarcoma by which they were affected and the type of circulating RNA detected. Methodology information included liquid biopsy type, assay used to investigate nucleic acid and, if applicable, the targets of the assay. Findings included the clinical use of the circulating RNA detected. Finally, the data sheet was reviewed and agreed by two senior authors (R.V. and G.M.) and two more authors (E.G. and M.B.B.) reviewed the results from the collected data. The initial literature search resulted in 243 studies. Once duplicates were removed and the articles were screened for inclusion and exclusion criteria, 83 studies remained and full texts were assessed for eligibility (Figure 1).

## 3. Results and Discussion

A total of 26 articles were included in this systematic review [34,35,36,37,38,39,40,41,42,43,44,45,46,47,48,49,50,51,52,53,54,55,56,57,58,59]. Of the 26 studies, 20 (77%) were retrospective and 6 (23%) were prospective studies. Quality scoring through the Methodological Index for Non-Randomized Studies (MINORS) [33] were then assessed:

- 15 (58%) were non-comparative studies and the mean score was 12/16 which represents a moderate quality;
- 11 (42%) were comparative studies and the mean score was 19/24 which represents a very high quality.

We evaluated 1381 patients. The mean age calculated on the population in the 16 articles where it was specified was 27 years old. Of these, 6 authors considered a pediatric population (<18 years) and the average age was 12 years, while 10 studies exclusively included the adult population (>18 years) and the average age was 39 years (Table 1).

Among all included patients, 991 (72%) were affected by bone sarcoma and 390 (28%) by soft tissue sarcoma. Specifically, among patients with BS: 783 (79%) osteosarcoma (OS) and 208 (21%) Ewing sarcoma (ES). Whereas, among patients with STS: 43 (11%) Kaposi sarcoma, 38 (10%) synovial sarcoma, 36 (9%) gastrointestinal stromal tumor (GIST), 19 (5%) rhabdomyosarcoma (RMS) and 15 (4%) myxofibrosarcoma (MFS). In 233 cases (61%), STS was referred to generically (Table 2).

By PCR-based methods, all the studies investigated circulating tumor RNA in the peripheral blood of the patients affected by sarcomas. Six papers (23%) have also studied the presence of tumor-derived RNA in bone marrow; in all of them were people with Ewing’s sarcoma. The studies investigated circulating tumor RNA, in particular the tumor expression and/or release of microRNA (miRNA) 11 (42%) studies, circular RNA (circRNA) 2 (8%) studies, long non-coding RNA (lncRNA) 1 (4%) study and, finally, counter-transcribed-RNA (ctRNA) 1 (4%) study. In 13 studies (50%), circulating tumor RNA was mentioned generically (Table 3).

The approach used to determine the tumor expression and/or release of RNA was either RT-PCR (11, 42%) or quantitative RT-PCR (qRT-PCR) (14, 54%). In a single study (1, 4%), droplet digital PCR (ddPCR) was used. According to the author’s finding, the circulating tumor-derived RNA has proven to have potential application in a clinical setting as/for disease detection and diagnosis (16), prognostic indicator (14), monitoring tumor dynamics such as disease progression/relapse (8), tracking the therapy response (7), and potential target therapy (5). In one work, a use as a “tumor characteriser” has emerged, which would allow differential diagnosis among STS. Furthermore, a single group of authors highlighted the potential correlation between malignant melanoma and Kaposi sarcoma through the detection of melanoma-associated markers in the peripheral blood of these latter patients. Several authors pointed out that this tumor-derived material can be potentially worthwhile in clinical practice in several areas at the same time. Finally, all authors seem to agree that further studies are needed to confirm each of these potential applications in clinical practice.

## 4. Discussion

Sarcomas are a large heterogeneous group of malignancies for which tissue biopsy represents the gold standard to make a diagnosis and to stage the disease [9]. Due to the limitations of this technique and in order to achieve early diagnosis, liquid-based biopsy techniques were developed [12]. Liquid biopsies are generally defined as those in which circulating cancer cell-derived components are detected in body fluids [13]. The field of circulating tumor nucleic acid detection, as a method of biology discovery or disease biomarker, has expanded rapidly over the past decade [41]. Bone sarcomas were the most represented in our sample, osteosarcoma in particular, followed by the heterogeneous group of soft tissue sarcomas. Assuming that cells show different expression patterns in cancer patients and healthy subjects, it was observed that circulating miRNA profiles of bone and soft tissue sarcomas are similar, despite the histological subtype, since both share a common mesenchymal origin and that serum miRNA profiles reflect the origins of human cancers [41].

In recent years, circulating non-coding RNAs are rising as key mediators in the regulation of gene expression of tumor cells profiles [60]. MiRNAs can regulate the expression of genes involved in the control of proliferation, angiogenesis and invasion [61]. Cancer cells release miRNAs into the extracellular surroundings, determining their presence in circulating body fluids where they remain highly stable [13]. These interesting properties suggest their potential use as blood-based markers in cancer diagnosis and prognosis prediction [62]. In this study, we have focused on the role of ctRNAs, such as circular RNAs, microRNAs and long non-coding RNAs, and their possible clinical application. Circulating tumor RNA levels can be related to sarcomas in either a direct or indirect proportional relationship, depending on whether they act as oncogenes or as cancer suppressors.

- ctRNA Prognostic Value

In 2017, Fujiwara T et al. performed a global miRNA screening in serum of OS patients, showing that the miR-25-3p expression levels at diagnosis revealed a significant difference in survival between patients with higher and lower expression levels of serum miR-25-3p, proving its prognostic value [47]. In 2021, Xie et al. also proved that circulating miR-26a-5p could be used as a diagnostic and prognostic biomarker for OS. Circulating miR-26a-5p levels in osteosarcoma patients was significantly higher than in healthy volunteers. Moreover, miR-26a-5p was significantly related to poorer overall survival and, in addition, they have shown its role as an independent risk factor for osteosarcoma in a multivariate analysis [35].

- ctRNA Monitoring Role

Other studies suggest that miRNAs may be used to monitor tumor dynamics such as disease progression and/or relapse. Indeed, it has been demonstrated that by measuring serum levels of certain miRNAs it was possible to monitor the tumor’s response to surgical resection by detecting any recurrence of disease at an early stage. In particular, it has been observed that specific miRNA levels were significantly reduced in OS patients after surgery [63]. In addition, low-serum miR-194 expression was strongly correlated with positive metastasis and advanced clinical stage.

Furthermore, serum miR-194 was confirmed to be an independent prognostic biomarker for osteosarcoma [39]. Circulating miRNAs also correlated with drug sensitivity. Kosela-Paterczyk H et al. studied a wide pattern of miRNA profiles; amongst these, they identified miR-140 to be associated with drug sensitivity in OS treated with doxorubicin, cisplatin and ifosfamide [40]. Other studies have shown that the expression of miR-21 in the serum of OS patients before and after chemotherapy correlated with its release in tumor tissues. In fact, in patients with effective chemotherapy, miR-21 serum levels decreased after treatment, suggesting that miR-21 can have a key role in monitoring the tumor response to treatment [64].

- ctRNA Diagnostic Value

A possible role of ctRNAs in differential diagnosis has also been studied. Serum miR-194 levels proved to have a good diagnostic value in identifying OS subjects and in differentiating them from both periostitis and healthy controls [39]. Moreover, Asano et al. showed that circulating serum miRNA profiles in sarcoma patients are clearly distinct from those in patients with other types of tumors. They developed Index VI, calculated from the levels of seven serum-circulating miRNAs, which has proven to be able to diagnose sarcoma accurately and to discriminate malignant tumors from benign tumors or healthy controls. Further, they tested the ability of Index VI in distinguishing the main histological subtypes of sarcoma: in bone sarcomas and malignant soft tissue tumors its accuracy was high, while in benign tumors it was low [41].

- Ewing Sarcoma and ctRNA: Liquid or Bone Marrow Biopsy?

Ewing sarcoma is one of the first sarcomas on which liquid biopsy was applied, taking advantage of the fact that it is characterized by the (11; 22) (q24; q12) chromosomal translocation that makes the EWS–ETS fusion gene through the transcription of EWS/HumFLIJ fusion RNA. The EWS–ETS gene is the driver of oncogenesis, and it is present in each tumor cell. EWS/HumFLIJ fusion RNA can be used as a highly specific target for RT-PCR. Because of that, ES is an excellent subject for this kind of technology, and all authors agreed that circulating tumor-derived RNA may be useful in detecting the disease and monitoring both tumor dynamics and response to therapy. Different papers have studied the use of EWS/HumFLIJ fusion RNA as a molecular marker for occult tumor cells in the peripheral blood and/or bone marrow of patients affected by ES. An association was shown between high level of EWS/HumFLI1 RNA and the presence of metastatic disease, which in turn correlates with a much more aggressive disease. Therefore, they suggest the application of RT-PCR for EWS/HumFLI1 RNA to help predict those patients who are destined for a poorer outcome [56]. Other authors, however, have questioned the usefulness of circulating material in the bloodstream compared to that found in BM. In fact, Ewing’s cells were detected by RT-PCR in BM and were associated with the presence of clinically detectable metastases and a statistically significant unfavorable outcome. Their findings suggested that the use of RT-PCR on BM but not on PB might be useful for the staging of patients with ES at diagnosis [55]. In 1995, intact tumor cells were the only source of positive RT-PCR results, since free RNA in the blood was not sufficiently stable to be detected by this method in the past [59]. With the introduction of digital droplet PCR (ddPCR), the sensitivity of circulating plasma tumor DNA (ptDNA), but also ptRNA, analysis has improved dramatically. However, it is costly, time-consuming and not yet available for widespread clinical use. Despite the use of this new technology, even in the most technologically sensitive assays, the detection of tumor-specific RNA from plasma in ES patients is quite unreliable, whereas the detection of tumor-derived DNA offers superior replicability as well as sensitivity [34].

- ctRNA as Target-to-Target Therapy

Furthermore, several authors have proposed miRNAs as the ultimate target of tailored therapies. In Ewing sarcoma, for example, EWS/HumFLIJ fusion RNA can be used as a highly specific target [56]. Similarly, miR-26a-5p, which can promote proliferation and migration of osteosarcoma by targeting HOXA5 acting as a tumor oncogene for osteosarcoma, could be exploited in the future as part of specific therapies for this disease [35]. Also, it was observed that the plasma level of lncRNA MT1JP was significantly lower in OS patients than in the control group. MT1JP overexpression inhibited the invasion and migration of OS cells by downregulating FGF2 through the upregulation of miR-646, which could directly target FGF2. Moreover, miR-646 inhibits cancer development by downregulating oncogenes, such as FOXK1 and EGFR. The authors therefore concluded that in the future both MT1JP and miR646 could be used as targets for specific therapies [38].

- ctRNA and Soft Tissue Sarcoma

Although in the literature there are more studies focusing on BS, the role of miRNAs was studied also for soft tissue sarcomas. High miR-1260b expression in serum was correlated with infiltrative MFS, for instance [30]. GISTs are the most common abdominal sarcoma associated with KIT or PDGFRA gene mutations. It has been demonstrated that overexpression of miR-196a is associated with a high-risk grade and metastatic disease, while miR-320a downregulation is associated with a shorter time to imatinib resistance [40].

- ctRNA and Its Use with Different Types of Cancer

Strong evidence about CTCs and ctDNA, but also ctRNA, as prognostic markers has been documented in many tumor entities including breast, prostate, lung and colorectal cancers in the past decades [65]. Erbes et al. proved that circulating miRNA can discriminate between patients with local breast cancer and healthy women [66]. In colorectal cancer (CRC), miRNA levels in peripheral blood were found to be upregulated in metastatic CRC, and therefore promising tools for diagnosis, prognosis, disease and therapy monitoring [16]. In lung cancer, specifically in patients affected by non-small-cell lung cancer (NSCLC), liquid biopsy made it possible to predict patients’ outcomes, especially for those who are not eligible for conventional biopsy [67,68].

- Liquid Biopsy, Pros and Cons

The plus of this approach, compared with tissue biopsy, is that it is minimally invasive, reproducible and free from potential complications [69]. Liquid biopsies have been shown to have the potential to detect material shed from multiple metastatic sites, rather than analyzing a small piece of tissue biopsy, to monitor cancer evolution in real time and to be capable of early detection of relapse [13].

However, when we speak about prognostic evaluation through liquid biopsy, we must take into consideration that not all patients have tumor cells detected by RT-PCR. There are many variables that may affect assay sensitivity negatively, such as sample preservation, RNA extraction methods, limited sensitivity of RT-PCR and analysis platforms [34]. When we consider BM as the source of our liquid biopsy, there could be also sampling error because involvement of the BM may be focal, so we may miss the involved sites, or, also, tumor cells may enter the circulation in an episodic way [56].

- Limits

This systematic review was limited by the differences between the articles with regard to study design, follow-up periods, outcomes and technology used that do not allow us to draw clear conclusions. Moreover, in the literature available today, there are few articles about ctRNA and its diagnostic, prognostic and monitoring roles in sarcoma. Few articles explicitly state what kind of function ctRNA can have and demonstrate it with solid statistical data. Most suggest a possible role that will need to be further investigated in the future. To the best of our knowledge, however, this is the first review in the literature to attempt to put together data specifically on ctRNA in patients affected by sarcoma in general with no age or specific diagnosis limits. Hereafter, it would be useful to standardize the investigation’s methods of circulating non-coding RNAs for each disease, in order to obtain more comparable data and therefore potential uses in the clinical field.

## 5. Conclusions

In conclusion, different studies proved the efficacy of specific ctRNA as blood-based markers in sarcoma for different purposes: diagnosis, monitoring and prognosis prediction. The single studies analyze specific ctRNAs in specific roles (diagnosis, monitoring, etc.), but few times have the results been confirmed by subsequent studies. This suggests that more studies are needed for its use in everyday clinical practice. The most consistent results between the different studies concern the RNA monitoring function. This is due to liquid biopsies’ capability to detect material cancer from multiple metastatic sites. In addition, ctRNA is today being studied as a target for molecular therapy and as a predictor of drug sensitivity; further studies are needed to deepen these specific uses. Ewing sarcoma turned out to be an excellent subject for this kind of technology due to its specific mutation, and all authors agreed that liquid biopsy may be useful in detecting and monitoring the disease. For staging of patients with ES at diagnosis, however, the findings suggested that the use of PCR on bone marrow is higher than its use on plasma. Liquid biopsy is particularly useful in patients not eligible for conventional biopsy, and also in patients affected by sarcomas.

## Figures and Tables

**Table 1:** Studies and demographic characteristics.

Authors	Year of Publication	Type of Article	MINORS		n° Patients	Mean Age (Years)
Bodlalk, A. et al. [34]	2021	Retrospective Study	non-comparative	12/16	5	13.6 *
Xie, X.Y. et al. [35]	2021	Retrospective Study	comparative	22/24	243	-
Morita, T. et al. [36]	2020	Retrospective Study	comparative	19/24	15	78
Yan, M. et al. [37]	2020	Retrospective Study	non-comparative	12/16	48	-
Yang, L. et al. [38]	2020	Retrospective Study	comparative	20/24	42	30
Shi, L. et al [39]	2020	Retrospective Study	comparative	20/24	124	-
Kosela-Paterczyk, H. et al. [40]	2020	Prospective Study	comparative	19/24	86	30
Asano, N. et al. [41]	2019	Retrospective Study	comparative	20/24	311	-
Piano Ma et al. [42]	2019	Retrospective Study	comparative	17/24	22	-
Kung-Peng, Z. et al. [43]	2018	Retrospective Study	comparative	16/24	50	-
Monterde-Cruz, L. et al. [44]	2018	Retrospective Study	comparative	19/24	15	20
Uotani, K. et al. [46]	2017	Retrospective Study	non-comparative	13/16	12	43.5
Wang, S.N. et al. [19]	2017	Prospective Study	non-comparative	15/16	102	-
Fujiwara, T. et al. [47]	2017	Retrospective Study	non-comparative	12/16	14	23
Allen-Rhoades, W. et al. [48]	2015	Retrospective Study	comparative	18/24	40	<18 *
Gallego, S. et al. [49]	2006	Retrospective Study	non-comparative	11/16	16	6 *
Yaniv, I. et al. [50]	2004	Retrospective Study	non-comparative	8/16	11	13 *
Palmieri, G. et al. [51]	2000	Retrospective Study	non-comparative	10/16	21	60
Wong, I.H. et al. [52]	2000	Prospective Study	comparative	18/24	11	21
Thomson, B. et al. [53]	1999	Retrospective Study	non-comparative	9/16	12	15 *
de Alva, E. et al. [54]	1998	Prospective Study	non-comparative	12/16	28	12 *
Fagnou, C. et al. [55]	1998	Retrospective Study	non-comparative	13/16	67	13 *
West, D.C. et al. [56]	1997	Retrospective Study	non-comparative	11/16	28	-
Peter, M. et al. [57]	1995	Retrospective Study	non-comparative	11/16	36	-
Pfleiderer, C. et al. [58]	1995	Prospective Study	non-comparative	12/16	16	-
Hamilton, R.F. et al. [59]	1993	Prospective Study	non-comparative	12/16	6	-
26		**RS (20)** **PS (6)**	**c (11)** **n-c (15)**	**19/24** **12/16**	**1381**	**<18–12** **>18–39**

MINORS—Methodological Index for Non-Randomized Studies; RS–Retrospective Study; PS—Prospective Study; c—comparative; n-c—non-comparative. * pediatric population.

**Table 2:** Primary tumor and sarcoma characterization.

Authors	BS		STS	STS Characterization
	OS	ES		
Bodlalk, A. et al. [34]	-	5	-	
Xie, X.Y. et al. [35]	243	-	-	
Morita, T. et al. [36]	-	-	15	Myxofibrosarcoma (15)
Yan, M. et al. [37]	48	-	-	
Yang, L. et al. [38]	42	-	-	
Shi, L. et al. [39]	124	-	-	
Kosela-Paterczyk, H. et al. [40]	16	8	62	Synovial sarcoma (26); GIST (36)
Asano, N. et al. [41]	78	-	233	not specified
Piano Ma et al. [42]	-	-	22	Kaposi sarcoma (22)
Kung-Peng, Z. et al. [43]	50	-	-	
Monterde-Cruz, L. et al. [44]	15	-	-	
Uotani, K. et al. [46]	-	-	12	Synovial sarcoma (12)
Wang, S.N., et al. [19]	102	-	-	
Fujiwara, T. et al. [47]	14	-	-	
Allen-Rhoades, W. et al. [48]	40	-	-	
Gallego, S. et al. [49]	-	-	16	Rhabdomyosarcoma (16)
Yaniv, I. et al. [50]	-	11	-	
Palmieri, G. et al. [51]	-	-	21	Kaposi sarcoma (21)
Wong, I.H. et al. [52]	11	-	-	
Thomson, B. et al. [53]	-	9	3	Alveolar rhabdomyosarcoma (3)
de Alva, E. et al. [54]	-	28	-	
Fagnou, C. et al. [55]	-	67	-	
West, D.C. et al. [56]	-	28	-	
Peter, M. et al. [57]	-	36	-	
Pfleiderer, C. et al. [58]	-	16	-	
Hamilton, R.F. et al. [59]	-	-	6	not specified
	**783**	**208**	**390**	

**Table 3:** Primary tumor and sarcoma characterization.

Authors	Primary Tumor	ctNAs	
Xie, X.Y. et al. [35]	OS	miRNA	miR-26a-5p/HOXA5
Yan, M. et al. [37]	OS	circRNA + miRNA	circPVT1/ miR526b/FOXC2 axis
Yang, L. et al. [38]	OS	lncRNA	lncRNAMT1JP
Shi, L. et al. [39]	OS	miRNA	miR-194/CDH2
Kung-Peng, Z. et al. [43]	OS	miRNA	hsa-circ-0081001
Monterde-Cruz, L. et al. [44]	OS	miRNA	miR-215-5p + miR642a-5p
Wang, S.N. et al. [19]	OS	circRNA	miR-491
Fujiwara, T. et al. [47]	OS	miRNA	miR-25-3p
Allen-Rhoades, W. et al. [48]	OS	miRNA	miR-214/LZTS1
Wong, I.H. et al. [52]	OS	ctRNA	COLL
Yaniv, I. et al. [50]	ES	ctRNA	EWS–FLI1
de Alva, E. et al. [54]	ES	ctRNA	EWS–FLI1 + EWS–ERG
Fagnou, C. et al. [55]	ES	ctRNA	EWS–FLI1 + EWS–ERG
West, D.C. et al. [56]	ES	ctRNA	EWS–FLI1
Peter, M. et al. [57]	ES	ctRNA	EWS–FLI1 + EWS–ERG
Pfleiderer, C. et al. [58]	ES	ctRNA	EWS–FLI1 + EWS–ERG
Bodlalk, A. et al. [34]	ES	ctRNA	EWS–FLI1 + EWS–ERG
Thomson, B. et al. [53]	ES+RMS	ctRNA	EWS–FLI1 + PAX3–FKHR
Gallego, S. et al. [49]	RMS	ctRNA	PAX7–FKH + PAX3–FKHR + AchR + MyoD1
Piano Ma et al. [42]	KS	miRNA	miR-375
Palmieri, G. et al. [51]	KS	ctRNA	MelanA/MART1 + Tyrosinase + p97
Uotani, K. et al. [46]	SS	miRNA	miR-92b-3p/Dkk3
Morita, T. et al. [36]	MFS	miRNA	miR-1260b/PCDH9
Hamilton, R.F. et al. [59]	STS	ctRNA	PCKβII
Kosela-Paterczyk, H. et al. [40]	OS + EW + SS + GIST	miRNA	miR-4772-5p + miR-582-5p + …
Asano, N. et al. [41]	OS + STS	miRNA	* 83 miRNAs

OS, osteosarcoma; ES, Ewing sarcoma; SS, synovial sarcoma; GIST, gastrointestinal stromal tumor; MFS, myxofibrosarcoma; KS, Kaposi sarcoma; STS, soft tissue sarcoma; RMS, rhabdomyosarcoma. * pediatric population.

## Data Availability

The datasets used and/or analyzed during the current study are available from the corresponding author on reasonable request.

## References

[1] Wunder J.S., Nielsen T.O., Maki R.G., O’Sullivan B., Alman B.A. (2007). Opportunities for improving the therapeutic ratio for patients with sarcoma. Lancet Oncol..

[2] Grünewald T.G., Alonso M., Avnet S., Banito A., Burdach S., Cidre-Aranaz F., Di Pompo G., Distel M., Dorado-Garcia H., Garcia-Castro J. (2020). Sarcoma treatment in the era of molecular medicine. EMBO Mol. Med..

[3] Surveillance, Epidemiology, and End Results (SEER) Program SEER*Stat Database: Incidence—SEER 9 Regs Research Data, Nov 2010 Sub (1973–2008)—Linked To County Attributes—Total U.S., 1969–2009 Counties, National Cancer Institute, DCCPS, Surveillance Research Program, Cancer Statistics Branch, released April 2011, Based on the November 2010 Submission. www.seer.cancer.gov.

[4] Cancer.gov https://www.cancer.gov/types/soft-tissue-sarcoma/hp.

[5] Burningham Z., Hashibe M., Spector L., Schiffman J.D. (2012). The epidemiology of sarcoma. Clin. Sarcoma Res..

[6] Ilié M., Hofman P. (2016). Pros: Can tissue biopsy be replaced by liquid biopsy?. Transl. Lung Cancer Res..

[7] Fulchignoni C., Cianni L., Matrangolo M.R., Cerrone M., Cavola F., Pataia E., Vitiello R., Maccauro G., Farsetti P., Rovere G. (2024). A Two-Step Approach to the Surgical Treatment of Soft-Tissue Sarcomas. Curr. Oncol..

[8] Perisano C., Vitiello R., Sgambato A., Greco T., Cianni L., Ragonesi G., Malara T., Maccauro G., Martini M. (2020). Evaluation of PD1 and PD-L1 expression in high-grade sarcomas of the limbs in the adults: Possible implications of immunotherapy. J. Biol. Regul. Homeost. Agents..

[9] Singh H.K., Kilpatrick S.E., Silverman J.F. (2004). Fine needle aspiration biopsy of soft tissue sarcomas: Utility and diagnostic challenges. Adv. Anat. Pathol..

[10] Eslami-S Z., Cortés-Hernández L.E., Cayrefourcq L., Alix-Panabières C. (2020). The Different Facets of Liquid Biopsy: A Kaleidoscopic View. Cold Spring Harb. Perspect. Med..

[11] Shah U.J., Alsulimani A., Ahmad F., Mathkor D.M., Alsaieedi A., Harakeh S., Nasiruddin M., Haque S. (2022). Bioplatforms in liquid biopsy: Advances in the techniques for isolation, characterization and clinical applications. Biotechnol. Genet. Eng. Rev..

[12] Nikanjam M., Kato S., Kurzrock R. (2022). Liquid biopsy: Current technology and clinical applications. J. Hematol. Oncol..

[13] Martins I., Ribeiro I.P., Jorge J., Gonçalves A.C., Sarmento-Ribeiro A.B., Melo J.B., Carreira I.M. (2021). Liquid Biopsies: Applications for Cancer Diagnosis and Monitoring. Genes.

[14] Gomes A.Q., Nolasco S., Soares H. (2013). Non-coding RNAs: Multi-tasking molecules in the cell. Int. J. Mol. Sci..

[15] Pan X., Xiong K., Anthon C., Hyttel P., Freude K.K., Jensen L.J., Gorodkin J. (2018). WebCircRNA: Classifying the Circular RNA Potential of Coding and Noncoding RNA. Genes.

[16] Kan C.M., Pei X.M., Yeung M.H.Y., Jin N., Ng S.S.M., Tsang H.F., Cho W.C.S., Yim A.K., Yu A.C., Wong S.C.C. (2023). Exploring the Role of Circulating Cell-Free RNA in the Development of Colorectal Cancer. Int. J. Mol. Sci..

[17] Botti G., Cantile M. (2018). Circulating long non-coding RNAs: Could they be a useful tool for cancer therapy monitoring?. Expert Rev. Anticancer Ther..

[18] Matsuzaki J., Ochiya T. (2020). Circulating microRNAs: Next-generation Cancer Detection. Keio J. Med..

[19] Wang S.N., Luo S., Liu C., Piao Z., Gou W., Wang Y., Guan W., Li Q., Zou H., Yang Z.Z. (2017). miR-491 Inhibits Osteosarcoma Lung Metastasis and Chemoresistance by Targeting αB-crystallin. Mol. Ther..

[20] Park E.G., Pyo S.J., Cui Y., Yoon S.H., Nam J.W. (2022). Tumor immune microenvironment lncRNAs. Brief. Bioinform..

[21] Li Z., Ruan Y., Zhang H., Shen Y., Li T., Xiao B. (2019). Tumor-suppressive circular RNAs: Mechanisms underlying their suppression of tumor occurrence and use as therapeutic targets. Cancer Sci..

[22] Botti G., Giordano A., Feroce F., De Chiara A.R., Cantile M. (2019). Noncoding RNAs as circulating biomarkers in osteosarcoma patients. J. Cell Physiol..

[23] Chellini L., Palombo R., Riccioni V., Paronetto M.P. (2022). Oncogenic Dysregulation of Circulating Noncoding RNAs: Novel Challenges and Opportunities in Sarcoma Diagnosis and Treatment. Cancers.

[24] Wei J., Liu X., Li T., Xing P., Zhang C., Yang J. (2020). The new horizon of liquid biopsy in sarcoma: The potential utility of circulating tumor nucleic acids. J. Cancer.

[25] Perakis S., Speicher M.R. (2017). Emerging concepts in liquid biopsies. BMC Med..

[26] Schwarzenbach H., Hoon D.S., Pantel K. (2011). Cell-free nucleic acids as biomarkers in cancer patients. Nat. Rev. Cancer.

[27] Lecomte T., Berger A., Zinzindohoué F., Micard S., Landi B., Blons H., Beaune P., Cugnenc P.-H., Laurent-Puig P. (2002). Detection of free-circulating tumor-associated DNA in plasma of colorectal cancer patients and its association with prognosis. Int. J. Cancer.

[28] Garcia-Murillas I., Schiavon G., Weigelt B., Ng C., Hrebien S., Cutts R.J., Cheang M., Osin P., Nerurkar A., Kozarewa I. (2015). Mutation tracking in circulating tumor DNA predicts relapse in early breast cancer. Sci. Transl. Med..

[29] Gray E.S., Schiavon G., Weigelt B., Ng C., Hrebien S., Cutts R.J., Cheang M., Osin P., Nerurkar A., Kozarewa I. (2015). Circulating tumor DNA to monitor treatment response and detect acquired resistance in patients with metastatic melanoma. Oncotarget..

[30] Min L., Bu F., Meng J., Liu X., Guo Q., Zhao L., Li Z., Li X., Zhu S., Zhang S. (2024). Circulating small extracellular vesicle RNA profiling for the detection of T1a stage colorectal cancer and precancerous advanced adenoma. Elife.

[31] Avsar M., Tambas M., Yalniz Z., Akdeniz D., Tuncer S.B., Kilic S., Sukruoglu Erdogan O., Ciftci R., Dagoglu N., Vatansever S. (2019). The expression level of fibulin-2 in the circulating RNA (ctRNA) of epithelial tumor cells of peripheral blood and tumor tissue of patients with metastatic lung cancer. Mol. Biol. Rep..

[32] Page M.J., McKenzie J.E., Bossuyt P.M., Boutron I., Hoffmann T.C., Mulrow C.D., Shamseer L., Tetzlaff J.M., Akl E.A., Brennan S.E. (2021). The PRISMA 2020 statement: An updated guideline for reporting systematic reviews. BMJ.

[33] Slim K., Nini E., Forestier D., Kwiatkowski F., Panis Y., Chipponi J. (2003). Methodological index for nonrandomized studies (minors): Development and validation of a new instrument. ANZ J. Surg..

[34] Bodlak A., Chang K., Channel J., Treece A.L., Donaldson N., Cost C.R., Garrington T.P., Greffe B., Luna-Fineman S., Sopfe J. (2021). Circulating Plasma Tumor DNA Is Superior to Plasma Tumor RNA Detection in Ewing Sarcoma Patients: ptDNA and ptRNA in Ewing Sarcoma. J. Mol. Diagn..

[35] Xie X.Y., Chen X.M., Shi L., Liu J.W. (2021). Increased expression of microRNA-26a-5p predicted a poor survival outcome in osteosarcoma patients: An observational study. Medicine.

[36] Morita T., Fujiwara T., Yoshida A., Uotani K., Kiyono M., Yokoo S., Hasei J., Kunisada T., Ozaki T. (2020). Clinical relevance and functional significance of cell-free microRNA-1260b expression profiles in infiltrative myxofibrosarcoma. Sci. Rep..

[37] Yan M., Gao H., Lv Z., Liu Y., Zhao S., Gong W., Liu W. (2020). Circular RNA PVT1 promotes metastasis via regulating miR-526b/FOXC2 signals in OS cells. J. Cell. Mol. Med..

[38] Yang L., Liu G., Xiao S., Wang L., Liu X., Tan Q., Li Z. (2020). Long Noncoding MT1JP Enhanced the Inhibitory Effects of miR-646 on FGF2 in Osteosarcoma. Cancer Biother. Radiopharm..

[39] Shi L., Xie C., Zhu J., Chen X. (2020). Downregulation of serum miR-194 predicts poor prognosis in osteosarcoma patients. Ann. Diagn. Pathol..

[40] Kosela-Paterczyk H., Paziewska A., Kulecka M., Balabas A., Kluska A., Dabrowska M., Piatkowska M., Zeber-Lubecka N., Ambrozkiewicz F., Karczmarski J. (2020). Signatures of circulating microRNA in four sarcoma subtypes. J. Cancer.

[41] Asano N., Matsuzaki J., Ichikawa M., Kawauchi J., Takizawa S., Aoki Y., Sakamoto H., Yoshida A., Kobayashi E., Tanzawa Y. (2019). A serum microRNA classifier for the diagnosis of sarcomas of various histological subtypes. Nat. Commun..

[42] Piano M.A., Gianesello L., Grassi A., Del Bianco P., Mattiolo A., Cattelan A.M., Sasset L., Zanovello P., Calabrò M.L. (2019). Circulating miRNA-375 as a potential novel biomarker for active Kaposi’s sarcoma in AIDS patients. J. Cell. Mol. Med..

[43] Kun-Peng Z., Chun-Lin Z., Jian-Ping H., Lei Z. (2018). A novel circulating hsa_circ_0081001 act as a potential biomarker for diagnosis and prognosis of osteosarcoma. Int. J. Biol. Sci..

[44] Monterde-Cruz L., Ramírez-Salazar E.G., Rico-Martínez G., Linares-González L.M., Guzmán-González R., Delgado-Cedillo E., Estrada-Villaseñor E., Valdés-Flores M., Velázquez-Cruz R., Hidalgo-Bravo A. (2018). Circulating miR-215-5p and miR-642a-5p as potential biomarker for diagnosis of osteosarcoma in Mexican population. Hum. Cell.

[45] Fricke A., Cimniak A.F.V., Ullrich P.V., Becherer C., Bickert C., Pfeifer D., Heinz J., Stark G.B., Bannasch H., Braig D. (2018). Whole blood miRNA expression analysis reveals miR-3613-3p as a potential biomarker for dedifferentiated liposarcoma. Cancer Biomark..

[46] Uotani K., Fujiwara T., Yoshida A., Iwata S., Morita T., Kiyono M., Yokoo S., Kunisada T., Takeda K., Hasei J. (2017). Circulating MicroRNA-92b-3p as a Novel Biomarker for Monitoring of Synovial Sarcoma. Sci. Rep..

[47] Fujiwara T., Uotani K., Yoshida A., Morita T., Nezu Y., Kobayashi E., Yoshida A., Uehara T., Omori T., Sugiu K. (2017). Clinical significance of circulating miR-25-3p as a novel diagnostic and prognostic biomarker in osteosarcoma. Oncotarget.

[48] Allen-Rhoades W., Kurenbekova L., Satterfield L., Parikh N., Fuja D., Shuck R.L., Rainusso N., Trucco M., Barkauskas D.A., Jo E. (2015). Cross-species identification of a plasma microRNA signature for detection, therapeutic monitoring, and prognosis in osteosarcoma. Cancer Med..

[49] Gallego S., Llort A., Roma J., Sabado C., Gros L., de Toledo J.S. (2006). Detection of bone marrow micrometastasis and microcirculating disease in rhabdomyosarcoma by a real-time RT-PCR assay. J. Cancer Res. Clin. Oncol..

[50] Yaniv I., Cohen I.J., Stein J., Zilberstein J., Liberzon E., Atlas O., Grunshpan A., Sverdlov Y., Ash S., Zaizov R. (2004). Tumor cells are present in stem cell harvests of Ewings sarcoma patients and their persistence following transplantation is associated with relapse. Pediatr. Blood Cancer..

[51] Palmieri G., Ascierto P.A., Satriano S.M., Strazzullo M., Apice G., Castello G. (2000). Circulating melanoma-associated markers detected by RT-PCR in patients with classic Kaposi’s sarcoma. Ann. Oncol. Off. J. Eur. Soc. Med. Oncol..

[52] Wong I.H., Chan A.T., Johnson P.J. (2000). Quantitative analysis of circulating tumor cells in peripheral blood of osteosarcoma patients using osteoblast-specific messenger RNA markers: A pilot study. Clin. Cancer Res..

[53] Thomson B., Hawkins D., Felgenhauer J., Radich J. (1999). RT-PCR evaluation of peripheral blood, bone marrow and peripheral blood stem cells in children and adolescents undergoing VACIME chemotherapy for Ewing’s sarcoma and alveolar rhabdomyosarcoma. Bone Marrow Transplant..

[54] de Alava E., Lozano M.D., Patiño A., Sierrasesúmaga L., Pardo-Mindán F.J. (1998). Ewing family tumors: Potential prognostic value of reverse-transcriptase polymerase chain reaction detection of minimal residual disease in peripheral blood samples. Diagn. Mol. Pathol..

[55] Fagnou C., Michon J., Peter M., Bernoux A., Oberlin O., Zucker J.M., Magdelenat H., Delattre O. (1998). Presence of tumor cells in bone marrow but not in blood is associated with adverse prognosis in patients with Ewing’s tumor. Société Française d’Oncologie Pédiatrique J. Clin. Oncol..

[56] West D.C., Grier H.E., Swallow M.M., Demetri G.D., Granowetter L., Sklar J. (1997). Detection of circulating tumor cells in patients with Ewing’s sarcoma and peripheral primitive neuroectodermal tumor. J. Clin. Oncol..

[57] Peter M., Magdelenat H., Michon J., Melot T., Oberlin O., Zucker J.M., Thomas G., Delattre O. (1995). Sensitive detection of occult Ewing’s cells by the reverse transcriptase-polymerase chain reaction. Br. J. Cancer.

[58] Pfleiderer C., Zoubek A., Gruber B., Kronberger M., Ambros P.F., Lion T., Fink F.M., Gadner H., Kovar H. (1995). Detection of tumour cells in peripheral blood and bone marrow from Ewing tumour patients by RT-PCR. Int. J. Cancer.

[59] Hamilton R.F., Vadhan-Raj S., Uthman M., Grey M., Holian A. (1993). The in vivo effects of PIXY321 therapy on human monocyte activity. J. Leukoc. Biol..

[60] Anfossi S., Babayan A., Pantel K., Calin G.A. (2018). Clinical utility of circulating non-coding RNAs—An update. Nat. Rev. Clin. Oncol..

[61] Baranwal S., Alahari S.K. (2010). miRNA control of tumor cell invasion and metastasis. Int. J. Cancer.

[62] Valihrach L., Androvic P., Kubista M. (2020). Circulating miRNA analysis for cancer diagnostics and therapy. Mol. Asp. Med..

[63] Zhou G., Lu M., Chen J., Li C., Zhang J., Shi X., Wu S. (2015). Identification of miR-199a-5p in serum as noninvasive biomarkers for detecting and monitoring osteosarcoma. Tumor Biol..

[64] Hua Y., Jin Z., Zhou F., Zhang Y.Q., Zhuang Y. (2017). The expression significance of serum MiR-21 in patients with osteosarcoma and its relationship with chemosensitivity. Eur. Rev. Med. Pharmacol. Sci..

[65] Alix-Panabières C., Pantel K. (2021). Liquid Biopsy: From Discovery to Clinical Application. Cancer Discov..

[66] Erbes T., Hirschfeld M., Rücker G., Jaeger M., Boas J., Iborra S., Mayer S., Gitsch G., Stickeler E. (2015). Feasibility of Urinary MicroRNA Detection in Breast Cancer Patients and Its Potential as an Innovative Non-Invasive Biomarker. BMC Cancer.

[67] Casagrande G.M.S., Silva M.O., Reis R.M., Leal L.F. (2023). Liquid Biopsy for Lung Cancer: Up-to-Date and Perspectives for Screening Programs. Int. J. Mol. Sci..

[68] Iqbal M.A., Arora S., Prakasam G., Calin G.A., Syed M.A. (2019). MicroRNA in lung cancer: Role, mechanisms, pathways and therapeutic relevance. Mol. Aspects Med..

[69] Ignatiadis M., Sledge G.W., Jeffrey S.S. (2021). Liquid biopsy enters the clinic—Implementation issues and future challenges. Nat. Rev. Clin. Oncol..

